# Brain disorders and the biological role of music

**DOI:** 10.1093/scan/nsu079

**Published:** 2014-06-19

**Authors:** Camilla N. Clark, Laura E. Downey, Jason D. Warren

**Affiliations:** Dementia Research Centre, UCL Institute of Neurology, University College London, London WC1N 3BG, UK

**Keywords:** music, emotion, evolution, mentalizing, dementia

## Abstract

Despite its evident universality and high social value, the ultimate biological role of music and its connection to brain disorders remain poorly understood. Recent findings from basic neuroscience have shed fresh light on these old problems. New insights provided by clinical neuroscience concerning the effects of brain disorders promise to be particularly valuable in uncovering the underlying cognitive and neural architecture of music and for assessing candidate accounts of the biological role of music. Here we advance a new model of the biological role of music in human evolution and the link to brain disorders, drawing on diverse lines of evidence derived from comparative ethology, cognitive neuropsychology and neuroimaging studies in the normal and the disordered brain. We propose that music evolved from the call signals of our hominid ancestors as a means mentally to rehearse and predict potentially costly, affectively laden social routines in surrogate, coded, low-cost form: essentially, a mechanism for transforming emotional mental states efficiently and adaptively into social signals. This biological role of music has its legacy today in the disordered processing of music and mental states that characterizes certain developmental and acquired clinical syndromes of brain network disintegration.

## INTRODUCTION

Music is universal and generally highly valued in human societies. These attributes are biologically grounded in the phylogenetically ancient neural machinery of emotion, reward and memory (Zatorre and Salimpoor, 2013). Young children reliably identify emotions expressed in music (Terwogt and Van Grinsven, 1988). As a means of emotional communication, music may have led language, even as our passions lead our reason (Rousseau, 1781). However, for neuroscientists no less than philosophers, the problem remains: why should these abstract auditory patterns, of no obvious contemporary biological value, be so powerfully embedded in the mental life and neurobiology of our species? A number of solutions have been proposed (Table 1), but previous accounts have not agreed a unifying evolutionary principle or, indeed, whether any such principle even exists (James, 1890; Dawkins, 1976; Patel, 2008).

**Table 1:** Proposed biological roles of music in human evolution: taxonomy of accounts

Author	Proposed primary biological role of music	Relationship to language	Cognitive and neural mechanisms
*Neural or cultural epiphenomenon*			
James, 1890	Incidental peculiarity of our nervous system	Incidental to language	By-product of language circuitry
Dawkins, 1976	Cultural communication via memes	Alternative memetic communication systems	Imitation circuitry
Pinker, 1997	Pleasurable epiphenomenon: ‘auditory cheesecake’	Parasitic on language	Co-opted generic processors (e.g. learning and reward)
Falk, 2004	Motherese facilitates speech processing; adaptive foraging and signalling strategy with remote infants	Prelinguistic vocal substrates for protolanguage with emergence of learned linguistic conventions	Prosody processing in tandem with speech capacity evolved in late australopithecines/early *Homo*
Jackendoff and Lerdahl, 2006	Elaboration of language faculty using an alternative symbolic code	‘Spandrel’ of language	Generic and domain-specific cognitive and neural (perceptual and affective) modules acting in concert
Patel, 2008	Emotional and aesthetic communication at cultural level, music-specific emotions	Analogous formal, categorical and combinatorial sound-based codes	Innate neural mechanisms process sound regularities modified by musical experience
*Courtship and other biological signals*			
Darwin, 1871	Courtship routines and territoriality	Pre-propositional musical protolanguage	Trans-specific vocal processing, imitative learning
Jespersen, 1922	Expression of instincts and strong emotions linked pre-eminently to courtship	Pre-cognitive protolanguage and training of speech organs by singing	Unspecified mechanisms mediating sexual selection
Miller, 2000	Courtship rituals and sexual selection	Language emerged from syntactic structures developed in musical vocal displays	Overlapping language and music areas in prefrontal and neocerebellar cortices
*Social bonding and cohesion*			
Brown, 2000a, b	Promotes group cooperation, coordination, cohesion of actions, thought and emotion expression	Homologous shared ancestor: ‘musilanguage’. Preferential processing for emotion (music) and referents (language)	Shared neuroanatomical substrates; stronger grounding of music in neurobiology and genetics
Cross, 2005	Social cohesion and cooperation and exploration of social behaviours with indeterminate outcomes	Alternative semantic systems varying in referential specificity	Orbitofrontal and limbic circuitry
Dunbar *et al.*, 2012	Enhanced coherence of social group and pair bonds, ‘vocal grooming’	Singing emerging first from vocal calls	Parasitized neocortical and neurochemical (e.g. endorphins)
Koelsch, 2011, 2013	Semanticized non-verbal communication code with extra-musical musicogenic meanings (emotion, intention), promoting social cohesion and strengthening inter-individual attachments	Continuum, music-primed language development via acoustic and structural similarities	Multimodal integrative, learning, social cognition and relative specificity from interaction of mechanisms
*Emotional signalling*			
Rousseau, 1781	Expression of strong emotions: love and hate	‘Passionate’ precursor to ‘rationality’ of language	Unspecified
Mithen, 2005	Long-range manipulation of others’ emotional states; from ‘motherese’, facilitated pair-bonds, social cohesion	Common prototypical ‘musi-language’ with subsequent divergence; pitch preceded rhythm and language	Mechanisms for vocal signal processing and some specialization for music
McDermott and Hauser, 2005	Aesthetic response to innate perceptual sensitivities and regularities; emotional communication	Alternative expressions of innate cognitive organization constrained by experience	General learning mechanisms driven by both neural and cultural factors, shaped by experience
Bharucha *et al.* 2006	Formal signalling code for emotion and mood regulation	Parallel non-propositional communication codes	Mechanisms for processing vocal emotion
Peretz, 2006	‘Education’ of emotions and auditory system derived from mother–infant communication	Unclear—potentially preceded or parallel	Partial specialization of cognitive and neural modules exposed by effects of brain damage with plasticity
*Vocal learning and non-verbal communication*			
Merker, 2000	Expressive mimesis and vocal learning	Key stage in vocal evolution leading to language	Perceptual, discriminative, attention, motor and learning
Fitch, 2006	Multiple selection pressures (e.g. sexual selection, infant caregiving and social cohesion)	Analogous formal system lacking semantic content	Innate mechanisms for complex vocal and hierarchical learning
*Pattern decoding and problem-solving*			
Huron, 2006	ITPRA model of musical expectation generating physiological responses, emotion and adaptive behaviours	Mutual interactions during evolution with formal analogies	Pattern processors linked to affective, neurochemical and autonomic adaptive mechanisms
Pressnitzer *et al.*, 2011	Rehearsal of emotional states minus painful outcomes, ambiguity resolution and exploration of alternate solutions	Intrinsic ambiguity of music in contrast to language may have promoted repeated exposure (listening)	Computational architecture of auditory scene analysis, schema-based perceptual and cognitive problem-solving
Zatorre and Salimpoor, 2013	Biological adaptation via reward-based emotion processing of predictable sound patterns generalizing to other kinds of stimuli	Common antecedents in vocal call sounds	Co-opted limbic, striatal (dopaminergic), autonomic reward circuits, linked perceptual and cognitive mechanisms
Juslin, 2013	Internal simulations of events that substitute for overt, risky actions	Divergence from common communicative system; music grounded in vocal emotion and semantic value in expectancies	Pattern analysis, meaning attribution and learning; problem-solving for ‘translation’ of musical ‘language’
Present account	Coding of potentially costly social routines for rehearsal, prediction and adaptation in surrogate low-cost form	Abstracted from call sound precursors in parallel, with diverging structural and semantic properties	Partly music-specific interaction of perceptual, cognitive, affective and autonomic mechanisms, critically exposed by brain damage and dysfunction

Representative accounts are presented and the table is organized according to the major theme of each account; these themes are inter-related and there is considerable overlap between accounts. ITPRA, imagination–tension–prediction–reaction–appraisal model (Huron, 2006)

Recent findings from cognitive neuroscience have shed new light on this old problem. Potential precursors to speech have been identified in geladas (Bergman, 2013) and hint at an evolutionary mechanism that may also be relevant to other modes of human social vocal behaviour, such as singing. Our emotional evaluation of music has been shown to depend on the dynamic interplay of multiple hierarchically organized brain mechanisms; these mechanisms are instantiated in distributed brain networks, including basal forebrain regions that encode biological drives and rewards, limbic regions that represent and evaluate emotional states, temporo-parietal cortical areas that represent structural harmonic and rhythmic properties of music, mesial temporal structures that support episodic memory and prefrontal areas that mediate psychological expectancy and social cognition processes (Huron, 2006; Downey *et al.*, 2013; Juslin, 2013; Koelsch, 2013; Salimpoor *et al.*, 2013; Zatorre and Salimpoor, 2013; Mas-Herrero *et al.*, 2014).

## A MUSIC BIOLOGICAL RATIONALE FOR STUDYING BRAIN DISORDERS

A further complementary approach to understanding the neurobiology of complex cognitive phenomena such as music is to assess neuropsychological effects of brain damage on those phenomena. This clinically focussed approach is neurobiologically compelling for two key reasons. First, in contrast to studies in the healthy brain, clinical studies (in general) identify neural substrates that are critical for function rather than merely epiphenomenal and delineate the relations between cognitive subprocesses (for example, by demonstrating that particular functions can dissociate). Second, the window opened by brain disorders on underlying cognitive architecture is particularly germane to the case of music, for which original biological functions have been largely obscured by the now dominant effects of cultural evolution. While we cannot rerun hominid evolution, the effects of brain damage can, in part, recapitulate the original evolutionary pathway. A substantial body of clinical data has now been amassed regarding the effects on music structural and emotion processing in developmental and acquired brain disorders, including autism (Allen *et al.*, 2009; Caria *et al.*, 2011; Allen *et al.*, 2013), stroke and other focal lesions (Jacome, 1984; Griffiths *et al.*, 2004; Satoh *et al.*, 2011), as well as neurodegenerative diseases (Drapeau *et al.*, 2009; Hailstone *et al.*, 2009; Omar *et al.*, 2011; Hsieh *et al.*, 2012; Downey *et al.*, 2013). These disorders target diverse brain systems including those mediating social cognition and theory of mind, semantic knowledge, emotion processing and biological reward. This clinical literature provides further evidence that musical deficits can be selectively linked to particular disease processes and that symbiotic, but separable cognitive and neural modules process music and language (Peretz, 2006).

## OUTLINE FOR A NEW MODEL OF THE BIOLOGICAL ROLE OF MUSIC

**Fig. 1:**
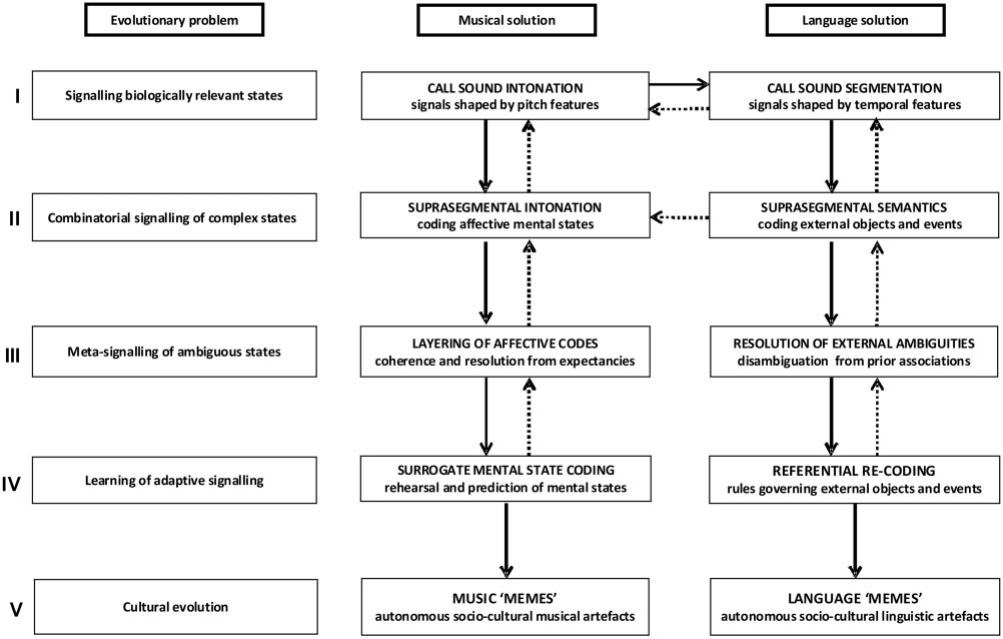
Proposed evolution of music as a code for transmitting surrogate mental states. The figure schematizes our model of the biological role of music in human evolution. Putative neurobiological problems that could have formed a basis for evolutionary selection are listed (left panels) together with proposed ‘solutions’ mediated by precursors of music (middle panels) and language (right panels), respectively. Although diagrammed here as a series of discrete ‘stages’ (I–V), we envisage the evolution of music as an essentially continuous process with successive stages, reciprocally influencing earlier processes as they became fully established (schematized here as reversible arrows) and increasingly abstract and autonomous coding at each stage; the final stage marks a transition from biological to cultural evolution that is arguably ‘irreversible’. In addition, we propose that earlier stages of music and language evolution shared processing mechanisms with increasing divergence at later stages. Our early primate ancestors may initially have used call sounds as vocal signals to convey to other members of the social group current states of immediate biological relevance (I), linking these with affective and perceptual brain mechanisms and establishing the earliest progenitors of music and speech through preferential use of pitch and temporal features, respectively. Extended ‘public’ vocal exchanges may have facilitated use of call sound sequences (II) for communicating more complex emotional states (proto-music) and objects and events in the environment (proto-speech), and ‘private’ off-line rehearsal of responses modulated by the listener’s own mental state. Combinatorial use of call sounds would, in turn, enable ‘meta-signalling’ of ambiguous emotional states and external phenomena (III) and resolution of these respective ambiguities through characteristically musical processes (e.g. harmonic expectancy) or language processes (e.g. association with prior object concepts). This meta-signalling capacity promoted the generation of emergent autonomous messages not closely tied to a particular mental state. Biologically and socially adaptive signalling (IV) for referential re-coding of objects and events in the world would then have entailed learning of language rules, whereas adaptive signalling for transmitting mental states engaged musical codes for rehearsing and predicting mental states in self and others. Stages I–III would have interacted cooperatively with development of an increasing capacity for mentalizing and ‘theory of mind’; music would then have been the most readily available vehicle for re-coding emotional mental states in surrogate form without engaging potentially costly social routines. Emergence of fully adaptive signalling would have enabled creation of musical and linguistic socio-cultural artefacts for autonomous transmission as ‘memes’ subject to cultural evolution.

Here we propose a new evolutionary model of music as a biologically sanctioned mechanism for transforming private, emotional mental states efficiently into public social signals. The model is outlined in Figure 1, alongside evolutionary biological ‘problems’ putatively ‘solved’ by each component of the model. We argue that music evolved (initially as ‘proto-music’) from the call signals of our hominid ancestors as a means to mentally rehearse potentially costly affectively laden social routines in surrogate coded form with high potential value, but low actual cost to hominids possessing the capacity. Implicit in our use of ‘costly’, here is the assumption that the physical, neural or emotional resources expended by actually engaging in the corresponding behavioural routines would have outweighed the resources invested in generating their proto-musical surrogates; we argue that this condition would have been met initially for high-stakes social scenarios such as mate selection, infant bonding, predatory threats and social separation. Vocal re-coding of such scenarios would have established adaptive linkages between brain mechanisms for processing biologically salient affective states and mechanisms for auditory signal analysis (Pressnitzer *et al.*, 2011; Zatorre and Salimpoor, 2013) and furthermore, this musical capacity may have evolved cooperatively with a capacity for interpreting mental states, mentalizing or ‘theory of mind’ (Downey *et al.*, 2013). We further argue that brain disorders expose the neuropsychological and neuroanatomical traces of this evolutionary role of music. We now present our model in detail, before considering how brain disorders inform the components of the model.

## KEY COMPONENTS OF THE MODEL

### Vocal signal processing

Call sounds are widely used by primates to convey emotionally laden conspecific information, particularly over long distances where visual cues are reduced (Ghazanfar and Santos, 2004); this information typically includes emotional states of immediate biological relevance (for example, proximity of danger, a food source or a mate; see Figure 1). Such ‘referential emotive vocalizations’ (Brown, 2000b) have both affective (internal emotional state) and semantic (external referent) specificity; these aspects may have been modulated primarily by pitch (intonation) and temporal features (call sound identity and segmentation). Non-human primates show some contextual flexibility of vocal communication (Tomasello and Call, 1997) to convey a relatively fine-grained repertoire of emotions and caller identity (Ghazanfar and Santos, 2004; Goodall, 2004). Certain primates combine call sounds to convey simple semantic messages (Arnold and Zuberbuhler, 2006) or gradations of feeling (Estes, 1992) and call sound concatenation might constitute an early precursor to the extended combinatorial vocal signalling of humans (Fitch, 2006a). Furthermore, certain non-human primates (notably marmosets) engage in ‘conversations’ with cooperative vocal turn-taking (Takahashi *et al.*, 2013). The essentially dynamic nature of sound allows primate callers to programme the temporal order in which key elements of the vocal message are presented. An enhanced capacity for vocal sequence production and processing in early hominids may have enabled blending of emotional content to represent more complex affective states, particularly via the medium of longer-timescale suprasegmental intonation patterns (see Figure 1). This stage may have been facilitated by more extended intimate vocal exchanges among bonded (e.g. mother–infant) dyads (Mithen, 2005). A pair-bonding context may have established the linkage between vocal precursors of music and dopaminergic, hormonal and other biological reward systems (Dunbar *et al.*, 2012; Salimpoor *et al.*, 2013). Furthermore, primate dyadic vocal exchanges are characterized by heightened pitch variation, rich harmonic content and reciprocity, as exemplified by human ‘motherese’ (Saint-Georges *et al.*, 2013) and gibbon songs (Koda *et al.*, 2013).

In line with previous suggestions (Mithen, 2005), we speculate that use of call sounds in this way may have consolidated a fundamental perceptual diversification of hominid vocal communication into primarily temporally segmented rhythmic ‘speech-like’ precursors (Bergman, 2013) and primarily intonational and harmonic ‘music-like’ precursors. This diversification has its contemporary legacy in the complex pitch and harmonic structures of music across human cultures, in contradistinction to the generally less pitchy, but precisely temporally structured architecture of most human languages (Patel, 2008). We do not wish to over-emphasize the evolutionary distinction between speech and music; certain persistent acoustic (Curtis and Bharucha, 2010; Bowling *et al.*, 2012) and neuroanatomical (Escoffier *et al.*, 2013) commonalities hint at an evolutionary trajectory that was at least partly shared. This is particularly true of music and prosody; however, whereas prosody has evolved primarily as an amplifier of linguistic content (Saint-Georges *et al.*, 2013), proto-music may have been better suited to code less hard-wired or ambiguous affective messages, such as those involved in the precarious rituals of pair-bonding and sexual foraging (Goodall, 2004).

### Public to private proto-musical signalling

This evolution of proto-musical vocal signalling may have been driven, in part, by an increasingly complex and flexible interaction between essentially ‘public’ (dyadic or group) and ‘private’ (own mental state) communicative functions. As elementary call sounds with conspecifically sanctioned, ‘public’ semantic value became linked into sequences modulated by caller emotional state in the context of primitive ‘conversations’, this would have promoted increasingly complex ‘off-line’ processing by the listener in preparing responses that were, in turn, modulated by the listener’s own emotional state. This off-line processing would, in turn, have set the stage for elaboration of fully autonomous privately rehearsed signals. While this public–private interaction of communication codes would have operated for proto-linguistic as well as proto-musical signals, it was proto-music (we argue) that, by virtue of its acoustic properties, governed coding of emotional states.

Facility in transitioning between public and private proto-musical signalling would have been socially and biologically advantageous both at an individual and group level because it would tend (for example) to promote empathy, pair bonding and social cohesion. This role of early musical signalling could, therefore, plausibly have been subject to evolutionary selection pressures. One might go further and propose certain ‘design features’ that these musical precursor codes were required to possess to be selected; these features would include not only repeatability and modularity (tending to promote efficient templating of shared codes by other individuals), but also more contentiously, sufficient ‘encryption’ to prevent uncontrolled access by all listeners (in particular, competitors) to the caller’s internal emotional states and potential vulnerabilities (Dunbar, 1999; Maynard Smith and Harper, 2003; Searcy and Nowicki, 2005). The proto-musical signalling we envisage would have guaranteed such access preferentially to listeners equipped and sufficiently motivated to engage in an active ‘code-breaking’ exchange: typically, the caller’s immediate kin or close social group. We speculate that key design features of modern music reflect these early evolutionary pressures (Fitch, 2006a). We also suggest that this evolutionary scenario would favour the development of maximally efficient rather than unnecessarily elaborate musical codes, which might explain both the unwonted power of ear worms and the comparative rarity in our music-loving species of the genetic constitutions exemplified by Mozart and Miles Davis.

### Meta-signalling and resolution of ambiguities

Increasingly sophisticated combinatorial use of call sounds by early hominids would have facilitated transmission of signals coding ambiguous states, whether instantiated in the world at large (proto-language) or in private emotional experience (proto-music). Successful resolution of such ambiguities would facilitate appropriate behavioural responses. We argue that the development of proto-linguistic and proto-musical ‘meta-signalling’ capacities provided a medium for representing and adaptively resolving apparent ambiguities and inconsistencies in the arena of the physical environment and social interactions, respectively. The scope of such meta-signalling would have extended to complex affective mental states accompanying social scenarios without immediate survival value; for example, those accompanying grief and mourning, social dominance or submission. Indeed, social situations routinely require abstraction of their inter-personal meaning and resolution of ambiguity if they are to be managed successfully; a prime instance of which is sarcasm (Kipps *et al.*, 2009).

Disambiguation of emotional states expressed in music may be based at least, in part, on learned associations about emotional coding derived from other sensory modalities (Gosselin *et al.*, 2007). However, rather than a single pre-eminent solution (as is typically required, for example, with perceptual ambiguities), adaptive resolution of novel, ambiguous emotional states (like those accompanying many social scenarios) may require their conflicting elements to be kept on-line and ‘harmonized’. Proto-musical signalling would have provided an evolutionary means to achieve this (see Figure 1). The forerunners of musical harmony would have enabled layering of disparate codes into a more complex message; pitch relationships create structure where, for other kinds of sounds, there would be cacophony and provide a means to resolve external disorder in a fundamentally predictable way. Abstract representation of blended, ambiguous or non-goal-directed emotional states or ‘floating intentionality’ is a feature of music today (Trost *et al.*, 2012). More particularly, resolution of ambiguity and release of musical tension based on established harmonic and other expectancies appears to underpin strong emotional responses (including ‘chills’) across musical genres (Huron, 2006; Salimpoor *et al.*, 2011, 2013). The ‘rules’ governing these expectancies are learned implicitly by members of the same musical culture (Tillmann, 2005).

The difficulty of aligning musical emotions with emotions expressed via other signalling channels (particularly vocal and facial expressions) has been advanced as an argument against a biological role of music (Allen *et al.*, 2013). However, certain analogies between music and other emotional channels have been demonstrated; there is convergence of emotional coding between musical and prosodic signals (Juslin and Laukka, 2003; Jackendoff and Lerdahl, 2006; Thompson *et al.*, 2012), while at least some musical emotions appear canonical between cultures (Fritz *et al.*, 2009). We argue that the very ambiguity and unclassifiability of musical emotions is close to the biological purpose of music. We propose that musical emotions are inherently difficult to classify because they are evolved to model the blendedness and ambiguity of our emotional reactions to our social milieu rather than merely recapitulating emotions conveyed more efficiently via other channels.

### Surrogate and predictive mental state coding

Development of a capacity for layering proto-musical codes might plausibly have facilitated the generation of ‘emergent’ messages not closely tied to a particular emotional state and ultimately, generation of such messages de novo. The coding of expectancies would further have enabled predictive signalling: a powerful means to establish proto-musical dialogues among group members and to allow the act of generating proto-music to influence the caller’s own rehearsed mental states reciprocally. We regard the emergence of such flexible autonomous signalling not bound to immediate emotional contingencies as a critical stage in the evolution of music, as it will have paved the way for use of musical signalling to code surrogate mental states (see Figure 1). We propose that this elaboration of musical vocal coding among our hominid ancestors evolved in tandem with (and reinforced) a capacity for interpreting the mental states of self and others: ‘theory of mind’ or ‘mentalizing’ (Frith and Frith, 2003). This capacity is multi-dimensional, including both cognitive (beliefs) and affective–perceptual (feeling states) components (Tager-Flusberg and Sullivan, 2000; Downey *et al.*, 2013). It is the affective–perceptual dimension that we emphasize here in respect to music. Music has been shown to model complex affective mental states such as ‘dreamy’, ‘adventurous’, ‘comforting’ or ‘seductive’ for healthy listeners (Downey *et al.*, 2013). This capacity is not contingent on previously learned associations, suggesting that coding psychological states is an important ‘indexical’ dimension of musical meaning that may parallel referential object associations in language (Koelsch, 2011). Understanding of agency in music (in common with other social cognitive functions) is mediated by a distributed anterior cortico-subcortical network (Frith and Frith, 2003; Zahn *et al.*, 2007; Steinbeis and Koelsch, 2009; Abu-Akel and Shamay-Tsoory, 2011).

A capacity for predictive signalling of surrogate mental states would have enabled emotional states to be rehearsed remotely from the corresponding experience in the world at large. This process would, in turn, have allowed evaluation of such states in recoded form for their biological cost and reward potential, both by the caller and by others in the social group. The young of many primate species have a protracted period of immaturity with substantial parental investment and extended opportunities for learning about and modelling the social environment (Tomasello and Call, 1997). Imitation and other forms of social learning appear to contribute to the establishment and differentiation of primate ‘cultures’ (Whiten *et al.*, 1999). Contemporary primate species engage in a variety of ‘play’ activities that rehearse essential behaviours such as fighting, mating and hunting. These activities enhance social behavioural flexibility and enable younger individuals to learn about their physical and social environments and their relative social standing within the group (Goodall, 2004; Palagi, 2006). Play detached from routine life activities has been proposed as an essential ingredient of human cultures (Huizinga, 1955; Nielsen, 2012). Vocal (including proto-musical) behaviours could plausibly have become similarly adapted in our own primate ancestors. The relevant ‘codes’ would be acquired, first, by closely associated members of the group (e.g. within dyads), before ultimately becoming adapted for use within the wider group. We envisage that initially this would have entailed reactivation of affective states recently experienced by particular callers. Later, however, musical codes may have come to signal affective states remotely experienced or never experienced by callers or listeners. Such independence would enable affectively laden social routines to be experienced in surrogate form within the social group. Essentially, proto-music would have become a cognitive tool with which to teach and vicariously experience the affective content of important, recurring social behaviours such as courtship, childrearing, grieving, social dominance and submission.

This feature of our model is in line with the notion that music enables internal simulations of events that substitute for overt risky actions (Juslin, 2013); here, we emphasize actions (behaviours) motivated by affective mental states. Sharing of mutually intelligible, proto-musical codes would allow highly arousing or potentially distressing emotional states experienced by particular members of the group to be managed within the group as a whole; much indeed as we still use music today (over scales ranging from private listening and intimate gatherings such as weddings and funerals to large-scale public events such as the September 11 memorial concerts). Furthermore, repeated shared rehearsals of musical codes within hominid groups would have maximized opportunities for exploration and refinement of alternative ‘solutions’ to the ‘problem’ resolved by the code. There are perhaps contemporary analogies here in listeners’ propensity repeatedly to seek variations on the same music (Pressnitzer *et al.*, 2011) or the on-line improvisatory exchanges of jazz ensembles.

Among primate species, the emotional states and behaviours around recurring social scenarios such as mate selection, aggression and bereavement are physiologically and psychologically expensive. These states are associated with potentially harmful neurohormonal, cardiovascular and other stress responses that can be modulated by music (Mostofsky *et al.*, 2013; Thoma *et al.*, 2013). The process of rehearsal would have consolidated the association of proto-musical codes with previously experienced arousing sensory experiences stored in memory; ultimately such rehearsal might have led to codification of a shared lexicon of schemas representing recurrent social routines, thereby further promoting social cohesion across the group (Pressnitzer *et al.*, 2011). Selection pressure for surrogate mental state encoding could have come from advantages for reproduction and survival conferred by rehearsing biologically significant, arousing mental states without the considerable potential cost of enacting them. As proto-musical vocal behaviours were already invested evolutionarily with high reward potential (Figure 1), individuals would be predisposed to engage in these behaviours, thereby facilitating opportunities for natural selection and ultimately, reinforcing the linkage with brain reward mechanisms. High arousal states engendered by music tend to be highly valued and to generate powerfully pleasurable responses (Salimpoor *et al.*, 2009), independently of affective valence. Among primate species, a preference for arousing music over less arousing auditory experiences appears to be a distinctively human predisposition (McDermott and Hauser, 2005). The pleasure we take in highly arousing, even sad, music may derive in part from experiencing this affective mental state without any actual correlate or cost to our wellbeing in the world at large (Allen *et al.*, 2013). Rehearsal of surrogate mental states is likely to have enhanced the individual’s capacity for empathy; this in turn, would have facilitated pair bonding, with advantages for mate selection and nurturing of offspring.

From a neuroanatomical perspective, mental state coding and interpretation engage a hierarchy of brain mechanisms, including those mediating affective value and biological reward (Abu-Akel and Shamay-Tsoory, 2011). Surrogate mental state attribution is therefore a plausible candidate to have recapitulated many of the component cognitive operations at work during the early evolution of music and this process of recapitulation is mirrored in a distributed neural circuitry. More speculatively, music may have a role in ‘repairing’ dysfunctional (though structurally intact) network elements and reintegrating emotional and cognitive processing in situations where these have become dislocated (Allen *et al.*, 2013).

### Music as a socio-cultural artefact

A substantial problem for all biological theories of music is the current status of music primarily as an art form (a socio-cultural artefact) with no obvious biological purpose. We do not of course wish to imply here that music is now solely a diversion or a means of generating pleasure. In most societies, (including the developed West) music continues to play a pervasive role in rituals, social cohesion and cooperative action among members of groups sharing a common musical culture (Koelsch, 2013). Rather, we argue that, in becoming abstract and autonomous, proto-musical communication codes became subject to cultural evolution as well as biological selection pressure and that over time, cultural evolution has become the primary force governing the development and uses of music within human societies. This process has also included the creation of other, non-vocal vehicles for conveying musical codes [in particular, musical instruments, which date from at least as early as the Neolithic period (Zhang *et al.*, 1999)]. The diverse varieties of music across cultures could be viewed (analogously with language diversity) as modus operandi for teaching locally agreed musical codes to a universal cognitive algorithm that transcends cultural boundaries (Fitch, 2006b). There are many examples of cultural evolutionary imperatives powerfully modulating or supplanting human biological imperatives (for example, ritual fasting, communal childrearing and voluntary celibacy). A useful shorthand here may be the concept of musical ‘memes’ (Dawkins, 1976; see Figure 1). While we acknowledge the limitations of this term, we use it here to emphasize the emergence of musical phenomena subject primarily to social and cultural rather than biological forces.

## HOW DO BRAIN DISORDERS INFORM THE MODEL?

**Fig. 2:**
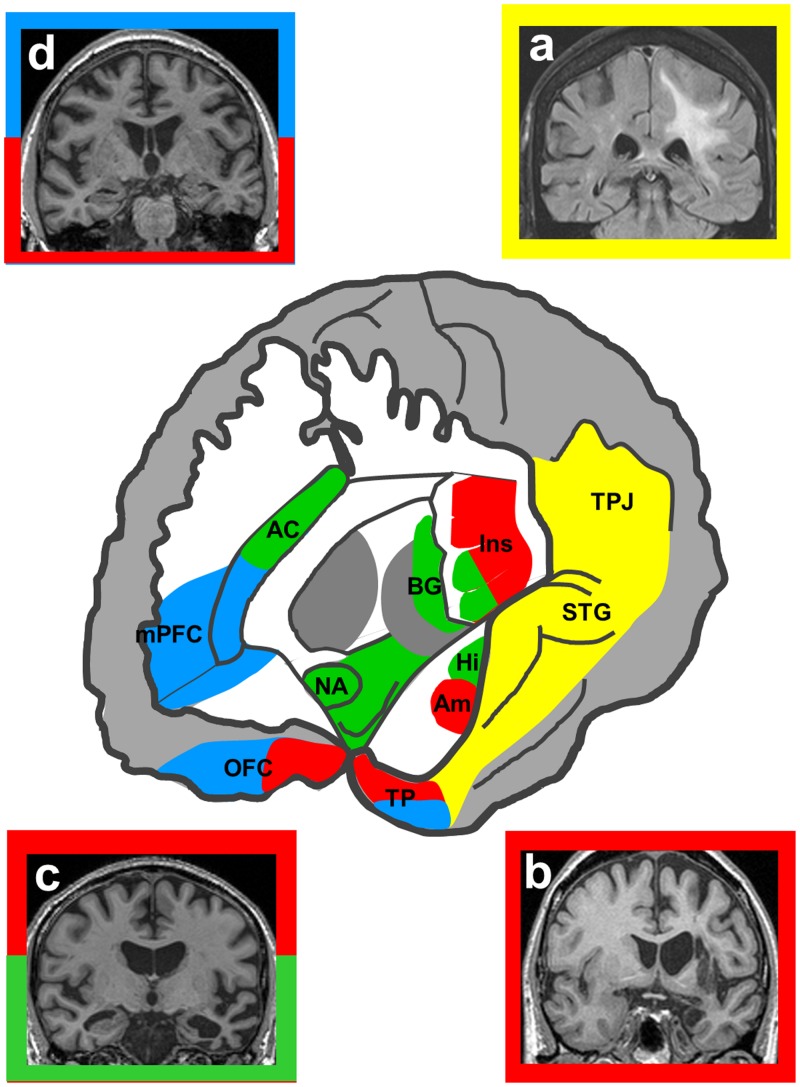
Neuroanatomy of music processing and effects of brain disorders. The central panel shows a schematic view of the brain dissected to reveal networks involved in music processing (the left hemisphere is projected forward here; however, relevant brain regions are bi-hemispherically distributed). Colours superimposed on the schematic code brain regions mediating broad cognitive operations underpinning music processing, based on normal functional imaging and clinical evidence. The primary cognitive operations associated with the regions are coded, as most regions are implicated in more than one operation (corresponding putative stages in the evolutionary model we proposed are numbered in parentheses, see Figure 1): yellow (I, II), perceptual analysis and imagery; green, biological motivation and reward encoding, autonomic responses (I, III); red, expectancies, associations and affective evaluation (III); blue, mental state processing and behavioural evaluation (IV). These operations are likely to be at least, in part, componential and hierarchically organized. Key: AC, anterior cingulate cortex; Am, amygdala; BG, basal ganglia; Hi, hippocampus; Ins, insula; mPFC, medial prefrontal cortex; NA, nucleus accumbens; OFC, orbitofrontal cortex; STG, superior temporal gyrus; TP, temporal pole; TPJ, temporo-parietal junction. The flanking panels show representative coronal brain sections from patients exhibiting abnormal music processing outlined according to the cognitive operations primarily implicated in that condition (the left hemisphere is displayed on the right in each case): (**a**) tumour involving temporo-parietal cortices and subcortical connections, associated with musical hallucinations; (**b**) infarction of insula and amygdala associated with selective loss of emotional response to music; (**c**) semantic dementia with focal, asymmetric anterior temporal lobe atrophy, associated with musicophilia and altered emotion coding in music; (**d**) frontotemporal dementia with selective bilateral frontal lobe atrophy associated with impaired ability to infer mental states from music and altered emotion coding in music. The scheme shown here complements the biological features presented in Table 2: each of these disorders (a–d) illustrates the componential neural architecture of music processing; (b) illustrates the effects of disrupted links with generic emotion processing mechanisms; (c) illustrates abnormal priming to particular musical codes; while (d) illustrates impaired modelling of surrogate mental states from music.

Evidence from brain disorders illuminates our model of the biological role of music in at least four major ways. We now discuss these with reference to the model as presented in Figure 1. Relevant illustrative clinical disorders are summarized in Table 2. Representative neuroanatomical profiles associated with particular brain disorders are shown in Figure 2, coded with the aspects of music processing that they putatively disrupt. The clinical evidence is underpinned by an overarching principle, the componential organization of music processing. This concept is illustrated by neuropsychological dissociations between competencies for music *vs* other complex cognitive phenomena (notably language) and among musical functions, observed in association both with focal brain lesions and neurodegenerative diseases (Rohrer *et al.*, 2006; Stewart *et al.*, 2006; Hailstone *et al.*, 2009; Omar *et al.*, 2010; Downey *et al.*, 2013). This fractionated organization argues for brain mechanisms that are relatively specialized for music and may therefore have evolved to process music or its evolutionary precursor.

**Table 2:** Brain disorders and the biological role of music

Music processing task^a^	Neuropsychological or behavioural deficit	Clinical associations	Neuroanatomical associations (see also Figure 2)
Elementary musical analysis and emotion processing (**I**)†	Selective deficits of musical scene analysis(Mazzoni *et al.*, 1993; McDonald, 2006), dissonance detection (Peretz *et al.*, 2001) and musical anhedonia (Griffiths *et al.*, 2004; Satoh *et al.*, 2011)	Focal lesions of either cerebral hemisphere	Particularly medial temporal and limbic structures, insula, auditory and temporoparietal cortices; links to subcortical reward circuits
Musical code processing (**II**)	Selective deficits of melody perception (Stewart *et al.*, 2006); excessive processing or priming for specific musical codes, e.g. musical hallucinosis (Griffiths, 2000) and musicophilia (Fletcher *et al.*, 2013)	Focal lesions of either cerebral hemisphere; diseases of ascending auditory pathways and neurotransmitter systems and focal neurodegenerative processes, especially semantic dementia	Deficits particularly with superior and anterior temporal, inferior frontal cortical damage; excessive processing with deafferentation/cholinergic deficiency in early auditory cortex, modulation of hippocampal interactions with distributed cortical networks
Musical meta-signalling: processing expectancies and associations (**III**)†	Altered processing of musical harmony, musical emotion associated with deficits or modulation of other channels of emotion processing (Peretz *et al.*, 1994; Bhatara *et al.*, 2009; Matthews *et al.*, 2009;Drapeau *et al.*, 2009; Gosselin *et al.*, 2007,^2011^; Caria *et al.*, 2011; Omar *et al.*, 2010,^2011^; Hsieh *et al.*, 2012)	Focal lesions and degenerations involving fontal and temporal lobes; developmental disorders, especially autism	Particularly anterior temporal and inferior frontal cortices and subcortical connections mediating emotional and semantic associations
Coding surrogate mental states (**IV**)¶	Specific deficit in attribution of affective mental states to music correlated with other social cognition deficits (Downey *et al.*, 2013), ‘rescue’ of social attributions by music (Bhatara *et al.*, 2009), correlation of musicality with social competence and emotional awareness (Ng *et al.*, 2013)	Developmental disorders, especially autism and Williams syndrome; focal neurodegenerative processes, especially frontotemporal dementia	Ventromedial prefrontal, anterior temporal cortices involved in mentalizing, frontoinsular projection neurons (Seeley *et al.*, 2006)

^a^corresponding putative stages in the evolutionary model we propose are indicated (in parentheses), see Figure 1; †evidence of linkage to non-musical processes of high neurobiological relevance; ¶evidence suggesting a specific neurobiological role of music or its precursors during human evolution. Overarching these lines of evidence is the componential organization of music processing, illustrated by neuropsychological dissociations between competencies for music *v* other complex cognitive phenomena (notably language) and among musical functions: this fractionated organization argues for brain mechanisms that are relatively specialized for music. Not indicated here are lesions that disrupt processing of music as a socio-cultural artefact (stage **V** of our model); for example, instrument apraxia and deficits of musical reading and writing

### Disorders of musical signal processing

A variety of selective deficits of elementary musical processing—i.e. deficits affecting the elements of music including spectrotemporal characteristics of musical sources and relations between sources—have been described following focal brain damage (Mazzoni *et al.*, 1993; Peretz *et al.*, 2001; McDonald, 2006; Stewart *et al.*, 2006). Such deficits may specifically impair emotional responses to music, producing ‘musical anhedonia’ (Griffiths *et al.*, 2004; Satoh *et al.*, 2011). Culprit lesions in such cases particularly involve medial temporal and limbic structures, insula, auditory and temporo-parietal cortices linked to distributed reward circuits. This circuitry is in proximity to the brain substrates of human voice processing (Hailstone *et al.*, 2011). Considered collectively, this evidence points to brain mechanisms that are at least relatively selective for musical signal processing, while at the same time preserving intimate functional and neuroanatomical relations with the neural mechanisms of human ‘call sound’ processing. The evidence further highlights a critical linkage between cortical mechanisms of musical pattern analysis and subcortical networks for processing reward and emotion (Pressnitzer *et al.*, 2011; Zatorre and Salimpoor, 2013).

### Disorders of musical code processing

Certain clinical phenomena demonstrate that the human brain is primed to rehearse and value particular musical ‘codes’; examples include abnormally enhanced, intrusive and repetitive musical imagery manifesting as ‘ear worms’ [pieces replayed in the mind’s ear (Sacks, 2007; Beaman and Williams, 2010)] or musical hallucinations [externalized percepts, particularly though not exclusively, occurring after cortical deafferentation in acquired deafness (Griffiths, 2000; Warren and Schott, 2006)]. These phenomena seem more likely to be driven by simple ‘catchy’ melodies than more complex pieces (Beaman and Williams, 2010). Even if the melodies are not reproduced out of memory, this autonomous auditory activity recapitulates structural features derived from musical experience (Warren and Schott, 2006). Heightened interest in and appreciation of music may attain the status of a specific, obsessional craving or ‘musicophilia’ in some clinical situations, including temporal lobe epilepsy, stroke, neurodegenerative disease and post-trauma (Jacome, 1984; Rohrer *et al.*, 2006; Sacks, 2007; Fletcher *et al.*, 2013).

### Disorders of musical meta-signalling

Altered processing of musical harmony and expectancies may follow focal brain damage (Peretz *et al.*, 1994), while impaired recognition of musical emotions (variably associated with deficits or modulation of other channels of emotion processing) has been described with focal lesions and degenerations involving the fontal and temporal lobes and developmental disorders, especially autism (Bhatara *et al.*, 2009; Matthews *et al.*, 2009; Drapeau *et al.*, 2009; Gosselin *et al.*, 2011; Caria *et al.*, 2011; Omar *et al.*, 2011; Hsieh *et al.*, 2012). This evidence supports a critical linkage between anterior temporal and inferior frontal cortices and subcortical networks mediating emotional and semantic associations, while at the same time allowing for a componential specificity in the coactivation of particular network components in response to music (Zatorre and Salimpoor, 2013). Within the domain of musical emotion, the ability to recognize specific emotions may dissociate from the general hedonic value of music (Matthews *et al.*, 2009; Omar *et al.*, 2012; Mas-Herrero *et al.*, 2014). This dissociation suggests that the emotional response to music itself has a componential architecture, as one might anticipate if the brain systems that process music evolved to link autonomic, affective and cognitive mechanisms over the course of human phylogeny.

### Disorders of musical mental state coding

Finally (and crucially for our model), brain disorders allow us to assess the extent to which music can model generic cognitive processes such as theory of mind. Frontotemporal dementia is the paradigmatic acquired disorder of human social behaviour (Seeley *et al.*, 2006; Kipps *et al.*, 2009). These patients are deficient in attributing affective mental states (but not non-mental representations) to music (Downey *et al.*, 2013). This deficit maps onto the previously proposed distinction between ‘indexical’ and ‘iconic’ dimensions of musical meaning (Koelsch, 2011). In addition, the deficit correlates with standard measures of social inference and empathy in patients’ everyday lives and has a neuroanatomical substrate in ventromedial prefrontal and anterior temporal lobe areas previously implicated in mentalizing and social concept representation (Frith and Frith, 2003; Zahn *et al.*, 2007, 2009; Steinbeis and Koelsch, 2009; Figure 2). In the face of often profound deficits of mentalizing and other aspects of social cognition, music appears to be an island of relatively preserved emotionality in autism (Allen *et al.*, 2009; Molnar-Szakacs and Heaton, 2012; Allen *et al.*, 2013). Music may even partly ‘rescue’ deficient social attributions by autistic individuals (Bhatara *et al.*, 2009). However, cognitive processing of musical emotions in autism may be quantitatively atypical and may be underpinned by altered engagement of ‘hub’ brain regions (in particular, anterior insula cortex) that integrate emotional responses (Caria *et al.*, 2011; Molnar-Szakacs and Heaton, 2012). Musicality correlates with emotional awareness and social competence in Williams syndrome (Ng *et al.*, 2013). Together, such studies present a prima facie case that mental state encoding was a key candidate function of music (or its precursors) during the evolution of the responsible brain systems. These disorders further delineate a distributed brain network that is critical for the modality-specific integration of emotions conveyed by music with other sensory affective channels, mentalizing and other processes involved in social cognition (Abu-Akel and Shamay-Tsoory, 2011; Omar *et al.*, 2011; Dowwney *et al.*, 2013). Work in frontotemporal dementia has demonstrated that the culprit network contains phylogenetically specialized neurons that are likely to support complex social behaviour (Seeley *et al.*, 2006).

## CONCLUSIONS AND FUTURE DIRECTIONS

Our model of music biology foregrounds predictive and adaptive decoding of patterned vocal signals and is, therefore, most closely aligned with previous accounts emphasizing a problem-solving or pattern-decoding function of music (see Table 1). However, the model incorporates elements that link other accounts emphasizing the playful potential of music (Pressnitzer *et al.*, 2011), its social significance (Darwin, 1871; Cross, 2005; McDermott and Hauser, 2005; Mithen, 2005; Bharucha *et al.*, 2006; Peretz, 2006; Patel, 2008; Koelsch, 2013) and role in vocal learning (Fitch, 2006a,b). Novel explanatory features of our model compared with previous accounts include the coding of private complex emotional mental states in surrogate form, and the potential insights held by clinical disorders of music processing into the componential neural architecture of music coding and mental state encoding. We have argued that a partly music-specific interaction of perceptual, cognitive, affective and autonomic mechanisms is critically exposed by effects of brain damage and dysfunction.

Our model suggests several avenues for future experimental evaluation. The vocalizations of other primate species could be compared quantitatively with human singing (Bergman, 2013). The limits of our capacity to rehearse mental states via music and the cognitive boundaries between music and other kind of vocal signalling could be assessed under ecological conditions by analysing the mentalizing properties of such special cases as whistled languages (Carreiras *et al.*, 2005), by comparing musical traditions and cultures beyond Western art music (Janata *et al.*, 2012) or perhaps by constructing artificial music (Charlton *et al.*, 2012) with specified neuropsychological properties. This, in turn, might allow formulation of a common trans-cultural lexicon of mental routines that can be modelled in music and their musical signifiers. In particular, by studying the effects of brain disorders, we can establish the extent to which brain systems are critical for music cognition, the degree to which more generic processes (such as theory of mind) are affected in tandem with music, the role of music in modulating such processes and their neurobiological substrates. Our clinical perspective leads us to assert that the pleasure we take in music may sugar the pill our brains once required to learn their social world.
